# Qualitative exploration of perspectives of the pharmacists working in public-sector hospitals during COVID-19 pandemic

**DOI:** 10.1186/s40545-023-00549-w

**Published:** 2023-03-17

**Authors:** Sitaram Khadka, Mohammad Saleem, Muhammad Usman, Furqan K. Hashmi, Fatima Tariq, Warda Zaheer, Sabahat Imon, Aqsa Inam, Ravi Prasad Gupta, Pallav Aryal

**Affiliations:** 1 Level One Plus Hospital, Golan Heights, Syria; 2 Shree Birendra Hospital, Nepalese Army Institute of Health Sciences, Kathmandu, Nepal; 3 Punjab University College of Pharmacy, University of the Punjab, Lahore, Pakistan; 4 Institute of Pharmaceutical Sciences, University of Veterinary and Animal Sciences, Lahore, Pakistan

**Keywords:** COVID-19, Global health, Pharmacists, Perception, Delivery of health care

## Abstract

**Background:**

The COVID-19 pandemic, a serious global health threat, has excruciating social and economic implications given its transmissibility, lack of therapy, and severity. In such a situation, pharmacists as frontline healthcare professionals hold a significant position to tackle. This study was designed to explore the perception and preparedness of pharmacists working in public sector hospitals amid such a pandemic in Pakistan.

**Methods:**

A total of 11 pharmacists were interviewed for this qualitative study design through a semi-structured interview guide. The interviews were recorded and transcribed verbatim.

**Results:**

The thematic content analysis yielded six major themes; understanding of COVID-19, perceptions towards COVID-19, preventive aspects, management aspects, changes to lifestyle, and psychological aspects. Though efficient preparedness and approach to fighting against such pandemics were reported, pharmacists were found susceptible to infection and psychological stress. They also expressed lockdown as an effective measure to prevent the disease from spreading but still were concerned about its economic and social impact.

**Conclusions:**

Adequate planning and facilities from the national level should be made available for strengthening the hospital pharmacy service that helps improve the overall healthcare system of low- and middle-income countries like Pakistan. The provision of a protective facility, incentives, and occupational health surveillance packages are deemed necessary to boost the self-esteem and morale of hospital pharmacists that safeguard the early and effective management of such disasters.

**Supplementary Information:**

The online version contains supplementary material available at 10.1186/s40545-023-00549-w.

## Background

COVID-19 continues to become one of the most serious public health challenges owing to its uncertainty of definite therapy. In low- and middle-income countries (LMICs) like Pakistan, healthcare services are under great pressure while responding to the pandemic. Along with other healthcare providers pharmacists globally are providing effective healthcare services amidst pandemics. Pharmacists are the first point of contact for seeking health care services owing to their accessibility and economic viability. People’s dependence on pharmacists increased significantly due to continuous pharmacy services even during lockdowns [1]. Pharmacists have a significant role during the pandemic in infection control as well as patient care and support through clinical and managerial roles [2–4]. Event-driven pharmaceutical care as well as uninterrupted supply chain management (SCM) of emergency medicines are the primary roles of hospital pharmacists in the management of the pandemic [5]. Pharmacists are consistently involved in COVID-19 management [6, 7]. Still, personal protection equipment (PPE) are somehow available for physicians, nurses, and paramedics, the pharmacists were least prioritized [1].

Healthcare professionals (HCPs) are at higher risk of developing mental health problems due to the increased workload, fear of infection, unavailability of adequate protective gear, and disruption in personal and social life owing to the lockdown. Pharmacists are no different. The exploration of the pharmacists’ perspectives on COVID-19, its management, and their preparedness is crucial as the information help concerned authorities develop some plan of action to address the issue in time.

To the best of our knowledge, this is the first of its kind endeavor to qualitatively explore the perception and preparedness of hospital pharmacists amid the COVID-19 pandemic in LMICs so that timely action could be taken.

## Methods

### Ethical approval

The research has been performed following the Declaration of Helsinki. The ethics approval was obtained from the Institutional Review Board (IRB)/Ethics Committee, University of the Punjab, Lahore, Pakistan (No: 50/DFEMS).

### Study design

Given the exploratory and flexible nature, qualitative research methodology was undertaken to focus on an in-depth understanding of the pharmacists’ perspective. In addition, qualitative analysis recognizes and fills in the gaps that are gone unobserved by the quantitative types [8]. Such methods are favorable for the analysis of amalgamate opinions, experiences, activities, and views of an extensive mix of respondents.

### Study setting, sampling, and inclusion criteria

The study was conducted in Punjab Province, the most developed and second-largest province of Pakistan. Pakistan falls under LMICs with over 212 million population fighting COVID-19 efficiently as per the World Health Organization (WHO) report [9]. According to recent data from the Punjab Pharmacy Council of Pakistan, there are 23,843 pharmacists in the province of Punjab [10]. The pharmacists-to-population ratio in Pakistan is 0.9:100,000 which is quite low compared to the WHO recommendation of 50:100,000 [11].

The participants in the study were selected based on their availability and willingness through the purposive sampling method. Participation in the study was completely voluntary and their identifications were kept confidential.

The targeted participants were registered pharmacy practitioners with at least a bachelor's degree in pharmacy and currently working in various public-sector hospitals of the Punjab Province, Pakistan as hospital pharmacists.

**Fig. 1:**
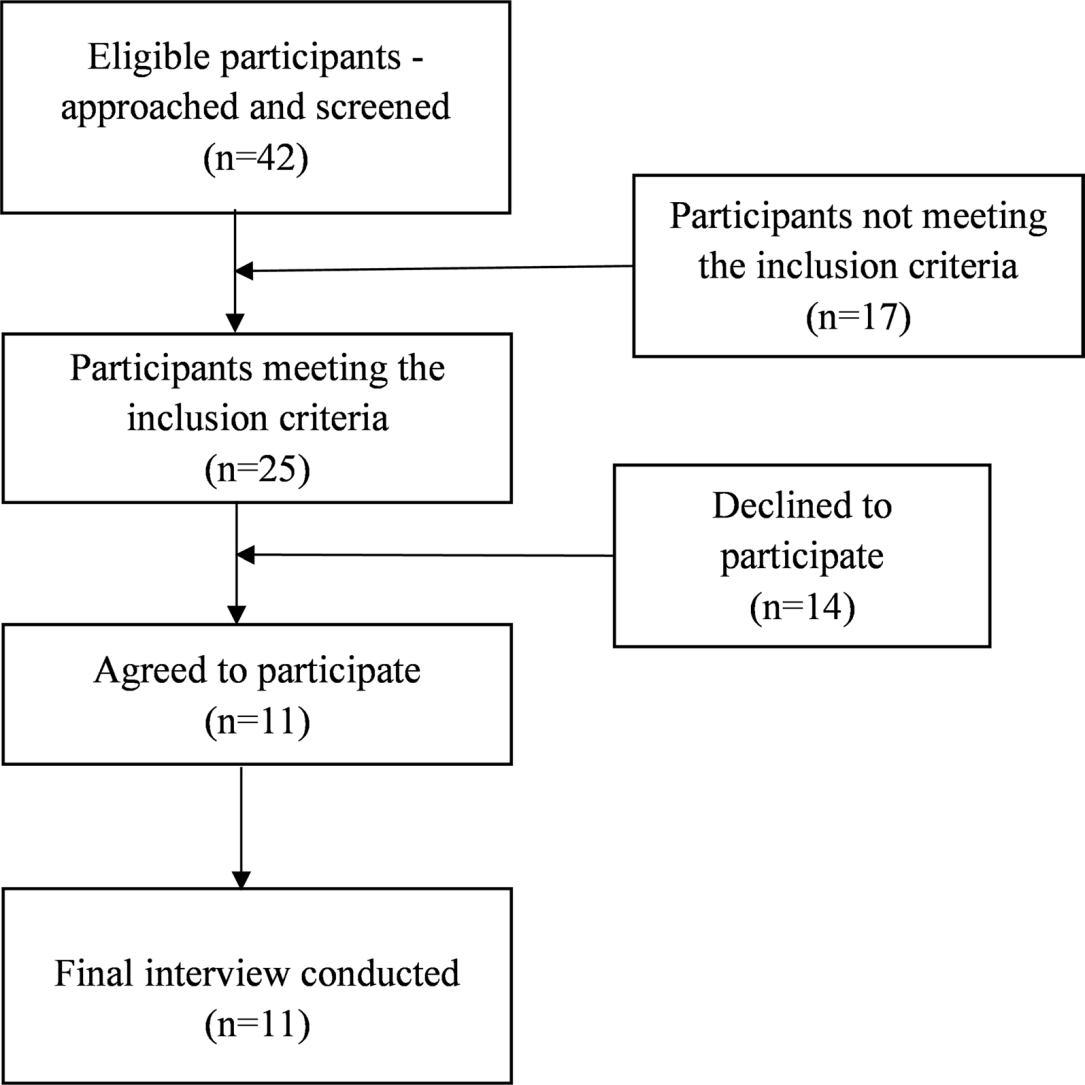
Flow diagram of the participants’ recruitment

A flow diagram of the participants’ recruitment is shown in Fig. 1.

### In-depth face-to-face interview

Based on the literature review and the current practices, a semi-structured interview guide (Additional file 1) was prepared and tested for face and content validity by two expert researchers at the Punjab University College of Pharmacy, Pakistan using cumulative and argumentative methods. It was pre-tested and verified for accuracy and consistency.

Verbal consent was taken by explaining the objectives of the study. The face-to-face interview was conducted with the participants at their workstations between March 2021 and May 2021 in the English language. Necessary probing was done to get more relevant information. The duration of each interview was approximately 20 to 30 min. All the interviews were audio-taped and transcribed verbatim. Data saturation was attained after 11 interviews, determining the final sample size of the study.

### Analysis

The data were thematically analyzed based on the method designated by Braun and Clarke by in-depth penetration into the interviews [12]. For analyzing qualitative data, this method provides a comprehensible as well as theoretically flexible line. All the audio-taped interviews were transcribed verbatim. Grammatical errors were corrected during the data extraction process. The research team embraced inductive and flexible methods for data collection. The identity of the respondents was kept confidential and the anonymity of opinions was also ensured by coding by the two male researchers (SK and MS). Transcripts were read repeatedly and data were analyzed manually. It was ensured that the generated codes and identified themes were rightly contemplative of the interview contents. Unanimity was reached among all the researchers in finalizing the result.

### Reporting

The COREQ (consolidated criteria for reporting qualitative research) checklist was utilized for reporting qualitative studies (Additional file 2) [13].

## Results

### Demographics of the participants

A total of 11 pharmacists (Phr-1 to Phr-11) were interviewed from eight different public sector hospitals. The majority of the respondents were female 72.73% (*n* = 8) with ages less than 30 years 63.64% (*n* = 7). About 81.82% (*n* = 9) of respondents were of under-graduate level qualifications. The majority of the respondents were involved in hospitals located in Lahore (*n* = 7) followed by hospitals from Sheikhupura (*n* = 2), whereas one respondent each was from hospitals at Shakargarh and Narowal (Table 1). All of them were actively involved in the service during the COVID-19 pandemic.

**Table 1:** Demographic characteristics of study participants

Characteristics	Parameters	Frequency (percentage)
Gender	Male	3 (27.27)
	Female	8 (72.73)
Age	≤ 30 years	4 (36.36)
	> 30 years	7 (63.64)
Education (Level)	Under-graduate (PharmD)	9 (81.82)
	Post-graduate (MPhil)	2 (18.18)
Placement	Lahore	7 (63.64)
	Sheikhupura	2 (18.18)
	Shakargarh	1 (9.09)
	Narowal	1 (9.09)

The demographic distribution of the participants is described in Table 1.

### Thematic content analysis

The thematic content analysis of the interview resulted in six major themes, Table 2.

**Table 2:** Thematic analysis of the interview data

Theme 1	Theme 2	Theme 3	Theme 4	Theme 5	Theme 6
1. Knowledge: understanding of COVID-19	2. Attitude: perception towards COVID-19	3. Practice – Prevention: preparedness for safety against COVID-19	4. Practice – Management: case assessment	5. Experience: barriers to lifestyle	6. Experience/outcome: psychological perspectives
1.1 COVID-19 transmission	2.1 Risk of getting infected with COVID-19	3.1 Preventive measures	4.1 Patient assessment	5.1 Impact of movement restriction and effect on daily activities	6.1 Self-esteem
1.2 COVID-19 management 1.2.1 Preventive measures 1.2.2 Therapeutic approach 1.2.3 Effectiveness of therapeutic approach	2.2 Lockdown approach-community level	3.2 Use of face masks	4.2 Tackling aspects (Self-assessment)-personal level		6.2 Stress
	2.3 Rules and guidelines	3.3 Vaccination hesitancy	4.3 Professional coordination		

#### Theme 1: familiarity with COVID-19

##### COVID-19 transmission

All the respondents considered COVID-19 as a respiratory infection that transmits through droplets from an infected person. The nose, eyes, hands, and face were regarded as the organs to take care of regarding COVID-19 transmission.

*“The most common way of transmission is through the air in the form of tiny droplets which are present in the breath of a patient suffering from coronavirus disease.” (Phr-9)*

##### COVID-19 management

All the respondents demonstrated proper concepts regarding COVID-19 management, such as supportive measures, repurposed drugs, immune boosters, vaccines, and definitive treatment.

*Preventive measures*: Answering a query regarding social distancing, hand washing, and using masks, gloves, and goggles as preventive measures; a majority of the respondents believed that such measures cannot totally prevent disease but only lower the transmission rate. They considered immune boosters as a better supportive measure for the prevention of COVID-19 transmission and therapy.

*“Social distancing, hand washing, using mask, gloves, and goggles can prevent from this disease.” (Phr-4)*

*“I think immune boosters are the best ways in order to protect ourselves from being infected by the virus.” (Phr-3)*

*Therapeutic approach*: The majority of the pharmacists highlighted supportive measures and repurposed drugs as the best potential treatment option. Similarly, all of them shared a similar view regarding the availability of vaccines and their efficacies.

*“All of the treatments going on are on hit and trial basis. The drugs which are being frequently used are azithromycin, hydroxychloroquine, tocilizumab, *etc*.” (Phr-2)*

*Effectiveness of therapeutic approach*: Regarding the effectiveness of ongoing supportive measures, the majority of the respondents insisted on patient compliance and their age as major factors. In such a scenario, they emphasized that the people must rely on the possible treatment options adopted for COVID-19 management if infected or became symptomatic with COVID-19.

*“These are all off-label use of medicines. We do not have any clinically proven evidence about the effectiveness of these medicines, but the therapy is ongoing in our practices.” (Phr-6).*

*“The therapy depends on the severity, immunity, and age of the patients because the response and symptoms vary accordingly.” (Phr-3)*

#### Theme 2: perception towards COVID-19

##### Risk of getting infected with COVID-19

All of the respondents, being front liners, considered that they are at substantial risk of contracting COVID-19 disease.

*“There are a lot of possibilities for the people who work in a frontline and our system lacks the facility to control infectious diseases. We cannot follow social distancing at our workplace.” (Phr-1).*

##### Lockdown approach—community level

Most of the participants demonstrated a positive response to the queries related to the lockdown approach. Whereas, some of them considered it a failure and cause of economic loss, and suggested the implementation of different standard operating procedures (SOPs).

*“It is a good action if people do follow it. It will be effective only after the support of every single person.” (Phr-11)*

*“Lockdown is basically to slow down the spread of the disease, not to curtail the disease in itself. It has a positive impact as far as we are concerned about the pandemic but, on the other hand, it has also economic, social, and other impacts that affect our daily life.” (Phr-7)*

##### Compliance with the rules and guidelines

All the respondents showed a similar type of positive reaction to the rules and guidelines set for the management and insisted that people should comply with the guidelines laid down by the government.

*“We need to cooperate with our government and healthcare system if we want to get rid of it. Trust your healthcare provider.” (Phr-2)*

#### Theme 3: preparedness for safety against COVID-19

##### Preventive measures

Almost the same responses were obtained regarding preventive measures adopted against the COVID-19 pandemic.

*“Before getting infected, I was not that much serious. But, now I try my best to keep on my face mask and gloves while dealing with serious patients and dispensing medicines. I am taking supplements, avoiding unnecessary contact with people like a handshake, hugging, and all that.” (Phr-3)*

*“We do hand washing, sanitizing, using a mask, and also social distancing” (Phr-6)*

##### Use of face masks

The majority of the respondents said that surgical face masks are more effective than cloth face masks. The majority of them suggested changing the mask every 2–3 h for effectiveness.

*“Surgical masks are 3-ply masks and are more effective.” (Phr-1)*

*“I think surgical masks should be changed after every 2 to 3 h or 2 to 3 times per day” (Phr-9)*

##### Vaccination hesitancy

The majority of the respondents were willing to get vaccinated. However, some of them were reluctant and showed interest only because there were no other options left.

*“Yes! If it shows sufficient safety data.” (Phr-11)*

*“Yes! We do not have any other choice.” (Phr-2)*

#### Theme 4: case assessment

##### Patient assessment

The majority of them had not directly assessed COVID-19 patients. Some of them handled COVID-19 patients in their ward visits with proper precautionary measures. Some respondents were limited to hospital pharmacies and their interaction was limited to caregivers instead of patients, whereas some pharmacists were mostly involved in medication management for COVID-19 patients.

*“The caretakers of COVID-19 patients visit the pharmacy on their behalf and take their medicines. So, I have not directly been in contact with such patients.” (Phr-2)*

*“I wear gloves before dispensing and sanitize my hands after dispensing. In case of in-patient prescription, we get the electronic prescription and we place medicines in bin and ward staff takes those for patients.” (Phr-3).*

##### Tackling aspects—personal level

All the pharmacists involved in this research were found to have tackled COVID-19 effectively following protocols; polymerase chain reaction (PCR) test, self-isolation, and supportive measures till the disease subsides. The same approach they would like to suggest to the patients as well.

*“I will self-isolate myself. I will practice proper hygiene and will take a standard regimen of treatment.” (Phr-6)*

*“I will quarantine myself for 14 days and then get tested again, if negative then get out of quarantine.” (Phr-10)*

##### Professional coordination

The willingness and practice to consult with medical doctors for medication shows inter-professional collaboration (IPC) and trust.

*“I will consult with a senior doctor for medication.” (Phr-10)*

#### Theme 5: barriers to the lifestyle

##### Impact of movement restriction and effect on daily activities

The lockdown approach and social distancing were kind of barriers to the everyday lifestyle during the COVID-19 pandemic though adopted as a containment strategy. Pharmacists were secluded from their families and friends. They missed the social gathering and outings and some of them were worried about the people below the poverty line.

*“I would rather say COVID-19 has turned life upside down. Our ways of living life, conducting ceremonies, and even ways of communication are changed altogether. We are going towards online seminars, ordering things online, and even patients are ordering medicines online nowadays. So, yes! There is a huge impact of the COVID-19 pandemic on our lives.” (Phr-7)*

*“Being a government sector pharmacist, I have not felt any difficulties except movement restriction, but many people are facing financial issues due to being jobless. The businesses collapsed and things are inflated. It is not easy for everybody even to buy the things like masks and sanitizers in Pakistan.” (Phr-2)*

#### Theme 6: psychological perspectives

##### Self-esteem

Most of the respondents did not feel lowered self-esteem. Though some of them thought that their self-esteem had slightly been lowered, all of them were optimistic and responded positively regarding the consequences of such lockdown and social distancing.

*“I think it does not affect our self-esteem as it is a reality nowadays and everyone knows about the severity of the disease and are now being serious towards its prevention.” (Phr-9)*

##### Stress

Mixed responses were obtained about stress related to the COVID-19 pandemic. The majority of the respondents, who were engaged in tough duty and were far from home reported being stressed. Some of them tested positive already and all of them were scared about getting infected. However, some respondents took this as an opportunity to enjoy themselves as they were getting some me-time and also getting to spend quality time with family.

*“I think, to some extent, this is enjoyable as it gives us free time and we can read books, watch movies, and can spend a good time with our family.” (Phr-9)*

*“It is not enjoyable for us because we have more duties, tiring and tougher duties.” (Phr-5)*

*“It is mentally stressful definitely. Staying away from home and family, not being able to stay in touch with friends, and the risk of getting infected is increasing stress but we are enjoying life as it is.” (Phr-10)*

## Discussion

The global threat created by COVID-19 and the lockdown measure adopted to contain the disease has changed the whole scenario of the world in all facets [14]. Pharmacists being drug experts and responsible HCPs are crucial components of the healthcare system owing to their clinical and managerial roles. The outcome of this study has exhibited a typical picture of pharmacy professionals as frontline healthcare practitioners amid the COVID-19 pandemic from the knowledge, perception, and attitude aspects.

A total of 11 pharmacists as respondents of our study were well-versed in the origin, causative agent, nature, and treatment strategy of COVID-19. The findings coincide with the results of different studies conducted globally [15–17] but differ from the findings of Bhagavathula et al. on overall HCPs, where most of the respondents had poor knowledge regarding symptoms, onset, and transmission of COVID-19 [18]. All the respondents perceived immune boosters such as vitamin C and zinc as effective preventive measures for mitigating COVID-19 and preventing its infection to some extent. Some of the respondents who were already infected with the virus found immune boosters very helpful in COVID-19 management. A review published by Khanna et al. also indicates that natural immune boosters are better supportive aids to fight against the COVID-19 pandemic [19].

The fear of getting infected and infecting others was found high among the respondents being frontline professionals who deal with COVID-19 cases directly or indirectly which concurred with many other studies [20, 21]. Lesser fear to be infected for self but higher to infect others was demonstrated by a study conducted in Jordan among HCPs which is comparable to the findings of our study [22].

The respondents were positive towards staying in isolation if tested positive or in quarantine if getting symptoms related to COVID-19 and following the medical advice of the clinician reports positive attitude and IPC among the HCPs as well. That is in line with the findings of various other studies among HCPs [23]. However, a study conducted in Northwest Ethiopia contrasts with such findings, where 90% of the HCPs were not willing to stay in isolation of the isolation centers [20]. The social stigma and psychological stress along with logistic issues may be the contributing factors to such attitude. Respondents’ optimistic view towards the lockdown as an appropriate approach for the management of pandemics but their concern towards the livelihood of people below the poverty line in such a scenario showed their professional as well as social integrity. To minimize the effect of lockdown on the socio-economic aspect they recommended a smart lockdown approach or development and implementation of SOPs to control infection without strict lockdown. Various studies advocated that the void between the theoretical research and the practical aspect of the implementation of lockdown has disregarded the consequential psychological effects [24, 25] and suggested for integration of the mental health aspect and good governance aspect in it.

Preventive measures; social distancing, hand washing or using sanitizer, wearing masks and gloves, and changing clothes frequently were adopted by all the respondents at an adequate level. Whereas, none of the respondents spoke about wearing personal protective equipment (PPE). Similar preventive measures were adopted globally by HCPs as shown by in different studies conducted around the world [26, 27]. In the present context of the unavailability of approved treatment, preventive measures are the best approach to limit the spread of coronavirus [28, 29]. Conversely, a study conducted in India reported the reluctance of some HCPs to adopt such measures [30].

Along with preventive measures, the regular mass testing of COVID-19, contact tracing, proper facility of quarantine and isolations, availability of hospital beds, intensive care units (ICU) capacities, number of ventilators, and oxygenation are crucial for COVID-19 management [31]. By providing a good working environment, such facilities enhance the morale and motivation of pharmacists and help gain ownership of the service. Support from the national level is expected in this regard for a better healthcare system.

Though some of the respondents were disinclined to get the vaccine, the majority were very keen. The respondents’ reluctance to get the vaccine implies their mistrust  on the immediate scientific development and fear of long-term adverse effects [32–34]. Different studies reported similar mixed types of responses from HCPs, where vaccine hesitancy, as well as willingness, were observed [32, 35–37]*.* A survey conducted among HCPs in France reported that 88.8% of pharmacists and 90% of physicians were willing to get vaccinated [38]. The scarcity of medical facilities may pose risk to the HCPs and ultimately to normal people. A multicenter study by An et al. also revealed the shortage of protective items such as masks to the HCPs that scared the HCPs [21] which indirectly affects patient care. Such healthcare facilities that lack many essential items, poor compliance towards treatment strategy, and weak prophylactic measures point towards requirement of  a national level of support towards education and awareness programs regarding healthcare services.

Most of the respondents perceived lockdown as an impediment to the normal routine and a barrier to a quality life. The isolation, workload, fear of getting infected and transferring to others, as well as social stigma, may lead to psychological stress and provocation of abnormal contemplations in them as highlighted by many studies [39, 40]. If such issues remain unaddressed that may lead to a disaster [41]. The majority of respondents’ firm self-esteem even at the time of the pandemic indicates an intact mental status. Contrary to this, many studies have reported psychological distress characterized by anxiety and depression among HCPs amid the COVID-19 pandemic [42, 43]. The respondents were found to be engaged in indoor activities as a coping strategy. WHO has identified proper health communication as a major coping strategy for a quality life amid the COVID-19 pandemic [44]. Adoption of an occupational health surveillance package with psychological interventions and preventive strategies to manage the psychological impact on HCPs including pharmacists helps manage such a pandemic [39, 44].

The compliance of the respondents towards the guidelines set forth by the government and the recommendation to comply with such guidelines indicates the integral nature of better healthcare service to manage the pandemic which is critical for the management of such a disaster.

Pharmacists have provided continuous service to patients despite the threat of the COVID-19 pandemic [45]. Pharmacists, in collaboration with other healthcare professionals and related stakeholders, can provide substantial contributions to the management of such pandemics. There is a need of strengthening the healthcare manpower such as pharmacists from the governing bodies, so that the integration of expertise in a united manner can have a significant impact on a better healthcare management system [46, 47].

### Limitations

The response failure and shortage of time due to the workload of the respondents and the COVID-19 issue can be considered some limitations linked to this study that affected the data collection. Furthermore, the findings may not be generalized to the whole country as this study was carried out only in the hospitals of Punjab province. However, the results in the other parts of the country would not be expected very differently.

## Conclusions

As frontline healthcare professionals, hospital pharmacists are equally prone to infection and psychological stress while working in such a situation of pandemics and the state of stigma. This calls for an immediate response from the concerned authorities to boost their morale and performance. It is observed that despite the humongous workload and mental distress attributed to restricted mobility and inadequate availability of medicines and related items, they are prepared to provide medical services complying with the set rules from the legislative bodies. This signifies the dedication, ownership, and intact willpower of hospital pharmacists to serve and fight the pandemic. By assessing their situations and challenges, this study provides support to the policy-makers to strengthen hospital pharmacy services that ultimately improve the healthcare system of low- and middle-income countries like Pakistan. It calls for the provision of a protective facility, incentives, and occupational health surveillance packages in healthcare settings that help maintain the self-esteem and motivation of hospital pharmacists, the indispensable component of the healthcare team, and facilitate the timely and effective management of such disasters.

## Supplementary Information

**Additional file 1.** Semi-structured interview guide.

**Additional file 2.** COREQ checklist.

## Data Availability

Data associated with this article can be obtained from the corresponding author upon reasonable request.

## Abbreviations

COVID-19: Coronavirus disease-2019
LMICs: Low- and middle-income countries
SCM: Supply chain management
PPE: Personal protection equipment
IRB: Institutional Review Board
WHO: World Health Organization
COREQ: Consolidated criteria for reporting qualitative research
SOPs: Standard operating procedures
IPC: Inter-professional collaboration
HPCs: Healthcare professionals
