# Supplementary material for: Qualitative exploration of perspectives of the pharmacists working in public-sector hospitals during COVID-19 pandemic
Source: J Pharm Policy Pract. 2023 Mar 17;16:45. doi: 10.1186/s40545-023-00549-w (PMC10022563; doi:10.1186/s40545-023-00549-w)
Supplement: Supplementary file 1 — Additional file 1. Semi-structured interview guide. [file 40545_2023_549_MOESM1_ESM.docx]

**Additional File**

**Qualitative exploration of perspectives of the pharmacists working in public-sector hospitals amid COVID-19 pandemic**

**Semi-structured interview guide**

**Part I**

**Focus: Knowledge about COVID-19**

1. What do you understand by COVID-19 Pandemic?
2. How does it get transmitted? What are the ways of its transmission? Does it get transmitted from human to human? What parts of the human body are more vulnerable to its transmission?
3. Are there any medications or vaccines approved for its treatment? Do you know any name of the drugs or treatment modality used for its therapy?
4. Do immune boosters help for COVID-19?
5. Who are at risk factors?

**Part II**

**Focus: Perception and attitude towards COVID-19**

1. How deadly is this disease?
2. Is there any possibility that you may get affected by the disease? If yes, in what way? If no, in what way?
3. Have you ever dispensed medicines to COVID-19 positive patients in hospital?
4. Have you ever assessed COVID-19 positive patients in hospital?
5. How do you dispense medicines if COVID-19 positive patients visits you in hospital?
6. How do you assess the COVID-19 patients?
7. Is social distancing, hand washing, using mask, gloves, and goggles prevent you from the disease?
8. Has this disease made any changes in your lifestyle?
9. Have you ever faced financial problem?
10. How often you go to hospital for duty? Is it regular like normal days?
11. How often you go out with from your residence besides duty time?
12. How do you take this lockdown for preventing the spread of COVID-19? Or it is not effective at all?
13. Do the medicines and treatment methods adopted till now can prevent this disease in an appropriate way? What are the pros and cons for this?
14. Are there enough measures in the area of your residing to cope with the pandemic? (community aspects)
15. Do you advise others about preventive measures for this disease? Why do you want to counsel others regarding this disease, to save yourself from getting it transmitted or to save the society from disease? (societal aspects)
16. Do cloth face mask functions the same way as surgical mask?
17. After how many hours there is a need to change face mask? What if people use the same mask for 2-3 days?
18. Is it must to sanitize hands with hand sanitizer or the use of just soap is enough?

**Part III**

**Focus: Preparedness for safety against COVID-19**

1. Are you well prepared to prevent yourself from this disease? If yes, what measures you are applying for this?
2. Do you often read the newspaper or watch videos or blogs related to COVID-19? If yes, do you follow the processes of safety described there?
3. What do you do if symptoms of flu that resemble COVID-19 occur?
4. What do you do if you were tested COVID-19 positive?
5. What type of hygienic conditions you are following? Hand washing (soap water/sanitizer/plain water), wearing mask/gloves/goggles while going out of the hostel, avoiding hugging/hand-shaking/ not touching the face by unclean hand?
6. How often you wash your hands/ take a shower/ change your clothes? Is there any specific reason to do so?
7. What is your view about vaccination?

**Part IV**

**Focus: Psychological perspectives**

1. Are you scared of getting infected with such a virus?
2. Is your self-esteem lowered due to this COVID-19 pandemic or due to lock down or both?
3. Is living inside the residence enjoyable to you? If yes, what are the ways you are applying for enjoyment? If no, what are the reasons for this, and what can be done to enhance the quality of life?
4. Are you missing gatherings and normal outings?
5. Is there any problem with lodging and food etc.?

**At last:**

Would you like to provide any additional comments about COVID-19, lockdown, and safety of the people?
